# European economies in terms of energy dependence

**DOI:** 10.1007/s11135-016-0350-1

**Published:** 2016-05-05

**Authors:** Anna Bluszcz

**Affiliations:** Faculty of Mining and Geology, Silesian University of Technology, 44-100 Gliwice ul. Akademicka 2a, Gliwice, Poland

**Keywords:** Energy dependence, Cluster analysis, k-means algorithm

## Abstract

The sustainable development and energy security are currently the priority challenges for the European Union countries. The sustainable and stable development of European economies is closely dependent on the stable access to energy resources. The constant increase of the demand for electricity requires long-term planning of the common European policy. The aim of the article is to analyse the fuel and energy resources situation of the member states with regard to their energy balances and with the determination of their import dependence in relation to fossil fuels, such as: coal, oil and natural gas. Based on the collected statistical data the analysis of clusters was presented in order to separate homogenous subsets, that is groups of the European Union Member States. The squared Euclidean distance has been adopted as the measure of similarities of the member states in the clusters, and the k-means algorithm has been used as the method of grouping. As a result of the analysis seven clusters were selected-groups of homogenous countries in terms of the import dependence due to the major energy resources (oil, natural gas and coal). The results of the paper can constitute bases for shaping the appropriate long-term common energy policy for the listed potential groups of countries. Statistical data were collected based on the Eurostat publication.

## Introduction

The sustainable development of European countries economies depends on the stable and permanent access to a variety of energy sources. Securing the reliable deliveries of the necessary minimum of energy in each country is a prerequisite for the security of the state and its citizens. The problem of energy security is an interdisciplinary concept that combines three perspectives at the same time, i.e., the energy, economic (market) and environmental perspective. The energy perspective includes the balancing of the demand and supply side, technical concept related to the technical infrastructure and its management and the diversification of deliveries of energy resources. The economic perspective is brought down to ensuring the acceptable price of energy resources, while the environmental one is related to ensuring the proper state of the natural environment for future generations.

Security of energy supply is closely related to micro- and macro-economic developments because imports and exports of energy may have an enormous impact on the balance of payments. Subsides, taxation and the costs or revenues of state-owned companies may have a considerable influence on the state budget. Moreover, the costs of energy are an important factor in the rate of inflation and in the international competitive position of a country’s economy (Correlje and van der Linde 2006; Jones et al. 2004; Knopf et al. 2015; Johansson 2013; EU 2008).

According to the International Energy Agency (2014) energy security is defines as the uninterrupted availability of energy sources at an affordable price. A standard definition of security of supply is a flow of energy supply to meet demand in a manner and at a price level that does not disrupt the course of the economy in an environmental sustainable manner. The concept is vast, multiform as it encompasses the whole physical and non-physical supply chain. It has also important time, space and social dimensions. It can be more precisely defined as:

- a reliable supply of energy. Choices both for primary energy sources and geographical suppliers ought to be as plentiful as possible, within a competitive framework, in order to reduce dependence on only one or two. Diversification in these two areas—primary energy sources and suppliers—is key to ensuring security of supply.
- A reliable transportation of supply. Transportation networks ought to be physically available to qualified players, well maintained, and expanded as required, and should offer as many competitive route options as possible.
- A reliable distribution and delivery of supply to the final customer. Energy ought to be efficiently delivered to the final customer according to particular time and quality standard without discrimination.
- At “reasonable price” over a continuous period. In theory, “reasonable” price means marginal cost reflective (Chevalier 2006).

Energy security has many aspects: long term energy security mainly deals with timely investments to supply energy in the line with economic developments and environmental needs. On the other hand, short-term energy security focuses on the ability of the energy system to react promptly to sudden changes in the supply–demand balance (IEA 2014). Furthermore, energy conversion and transport are also mentioned in relation to energy security as disruptions can occur anywhere in the supply chain (Jenny 2007; Scheepers et al. 2007) or the ability of the system to cope with extreme events, such as hurricanes, strikes, terrorist actions and lastly, the political stability of supplying and transit countries appears in discussions (Jansen et al. 2004; Le Cog and Paltseva 2009).

Generally, the energy security means the state of the economy than enables the production of energy, ensuring the stable deliveries and complying with the environmental requirements. Another definition of energy security means the analysis of the state and possibilities of producing energy and anticipating disruptions of this process (Probierz 2015).

Achieving energy security requires efforts to reduce risks to energy systems, both internal and external, and to build resilience for managing the risks that remain. Tools to achieve this include: ensuring markets function so that demand and supply meet optimally; providing adequate production and transport infrastructure; developing risk management systems (reserves, emergency planning and alternative supply routes); maintaining a diversified portfolio of energy suppliers; and keeping demand under control (energy efficiency). Energy security considerations must also be weighed against economic competitiveness considerations and environmental concerns (ISSUE 2014).

In the case of the European Union we have to deal with a particular understanding of the energy security, which consists of many elements. Energy security is usually presented as the element of the energy policy, hence we often deal with the intertwining of the concepts of energy security and energy policy. The starting point for understanding the energy policy of the European Union is the security of deliveries– in the traditional approach. First, the problem of securing the energy deliveries was associated with the possibility to meet the demand for energy generated by the state, however, this approach proved to be too limited. Therefore, the security of energy deliveries has been associated with the security on the social and economic level, what is confirmed by the definition of the security of deliveries included in the green paper, which means ensuring, for the well-being of the society, the efficient functioning of the economy of the physically continuous availability of energy products on the market, at prices available for all consumers (private and industrial) while taking into account the concern for the state of the environment and sustainable development (Green Paper 2001). Subsequently, the concept of security of deliveries has been expanded to include the following elements: a competitive market, new technologies, infrastructure. Without competition and technical possibilities of delivery and acceptance of energy it is not possible to complete the homogenous energy market, which is the expression of the progressive integration of the EU member states. Within the EU, the construction of the single market faces problems related to the insufficient energy infrastructure. This results from many factors, among others, energy structures of the member states, political and economic interests, positions of the energy companies, high costs of the energy infrastructure. Therefore, it is important to allow the construction of the single transmission network, which will enhance both the security of deliveries and the competitiveness. The lack of possibilities to become independence from the EU from the import of energy carriers has impacted the expansion of the energy policy with the issue of demand management. Hence it is important to undertake directions of activities, which are to reduce the risk of dependence, i.e., the support for the development of new technologies, support for the renewable energy sources, diversification of energy carriers. These elements are reflected in the regulations of the energy and climate package and Energy Roadmap 2050 (Energy Roadmap 2015).

Against the background of the broadly outlined issues of the energy security, which is closely related to the economic zone, the main aims of the article were clarified, which include: the analysis of the energy market of the member states with the particular focus on diversification of the member states in terms of their energy mix and the construction of the grouping model of countries into homogenous groups in terms of their level of energy dependence in terms of gas, oil and coal concentration. The results of the presented analysis can serve as the basis for creating long-term energy policies common to a given group of countries, taking into account the specific and diversified conditions of the member states grouped in the grasp according to the statistically selected homogenous clusters. The square of the Euclidean distance has been adopted as the measure of similarity of the member states in the clusters, and the k-means algorithm has been used as the grouping method.

Due to the fact that the main energy resources for the electricity production in the world include the fossil fuels, i.e., oil, coal and natural gas, and their dominant role of the market of energy resources according to the projections will persist for at least the next two decades. In the article the data on levels of dependence of the member states in terms of these three main energy resources has been adopted for analysis. And so, e.g., in 2012 oil accounted for as much as 33 % of the world consumption, the next place was taken by carbonaceous fuels constituting 30 % and the third place goes to natural gas, constituting 24 % of the global consumption. While the nuclear energy constituted only 4 %, and the renewable energy 9 % of the world consumption (Dreyer and Stang 2014).

## The analysis of the energy market of the European Union Member States

The structure of electricity production in the European Union countries differs from the world structure mainly due to the significantly lower level of fossil fuels use to produce electricity, which currently in the EU constitute only 45.8 % i.e. about 41 % less than in the general world structure. The use of nuclear energy in the EU currently constitutes 12.5 %, water energy 20 %, and renewable energy sources as much as 21.7 %. Scenarios for the development of energy systems in the European Union countries in 2030 assume, among others, the decline in the share of fossil fuels for the electricity production to the level of 39 %, the increase in the share of nuclear energy to the level of 22 %, the increase in the share of renewable energy resources to 26 % and the decline in the share of water energy to 13 % (Clerens et al. 2015; Szczerbowski 2015).

The European Union is striving to be the leader in implementing renewable energy solutions as main sources of electricity in an effort to overcome the greenhouse gas (GHG) problem achieving a low carbon electricity sector in line with global concerns regarding the environmental issues caused by the greenhouse effect (Franki and Viskovic 2015; Boluk and Mert 2014; Stern 2008). This situation stems from the 2030 goals adopted by the Council of Europe in terms of: 40 % reduction in greenhouse gas emissions compared to 1990 levels, 27 % of energy consumption to come from renewable sources, 27 % improvement in energy efficiency (EC 2013; FEA 2016; Carvalho 2012; Burman et al. 2014). At present, the electricity sector is the single largest contributor to GHG emissions. It is, therefore, also expected that this sector will have to be called upon to carry the biggest load when the required reductions in emissions are concerned requiring the adoption of low-carbon generation technologies (Grubb et al. 2008). This fast expansion of renewable energies, particularly in the electricity sector, is supported by many initiatives on the European and governmental level, including the direct economic support. In the 2007–2013 financial perspective, nearly 11 billion of Euro, representing approx. 3 % of the EU general budget, was allocated to the energy purposes such as: development of renewable energy sources, increase of energy efficiency and construction of trans-European networks (Pająk and Mazurkiewicz 2014).

Total production of primary energy for the EU-28 was 789.7 million tonnes of oil equivalent (toe) in 2013. The EU-28’s major primary energy producers were France (17.1 %), Germany (15.3 %) the United Kingdom (13.9 %) followed by Poland (8.9 %) and Netherlands (8.8 %) what was presented in Table 1. It is important to note that in the 2004–2013 decade the United Kingdom has reduced its primary energy production by more than 50 %. In 2013, 12 EU Member States decreased their energy production EU while the rest increased it.

**Table 1:** Total production of primary Energy (Mto_e_) in EU Member States in 2013

EU-28	Total production (Mto_e_) 789.7	Share of percentage 100 %
France	135.1	17.1
Germany	120.6	15.3
United Kingdom	109.5	13.9
Poland	70.6	8.9
Netherlands	69.7	8.8
Italy	36.9	4.7
Sweden	34.7	4.4
Spain	34.3	4.3
Czech Republic	29.9	3.8
Romania	26.1	3.3
Finland	18	2.3
Denmark	16.6	2.1
Belgium	14.6	1.8
Austria	12.1	1.5
Bulgaria	10.5	1.3
Hungary	10.1	1.3
Greece	9.3	1.2
Slovakia	6.4	0.8
Portugal	5.8	0.7
Estonia	5.7	0.7
Croatia	3.6	0.5
Slovenia	3.6	0.5
Ireland	2.3	0.3
Latvia	2.1	0.3
Lithuania	1.4	0.2
Cyprus	0.1	0.0
Luxembourg	0.1	0.0
Malta	0	0.0

*Source* Eurostat, statistical books: Energy, transport and environment indicators (2015) edition

Primary energy production from solid fuels accounted for 80.50 % in Poland, 78.3 % in Estonia, 72.3 % in Greece and 59 % in the Czech Republic. Crude oil was used at a very low percentage by the majority of EU Member States except Denmark (53.3 %), The United Kingdom (36.1 %), Romania (15.9 %), Italy (15.2 %) and Croatia (15.0 %). Natural gas was widely used for the production of primary energy mainly in the Netherlands (88.7 %), Croatia (41.6 %), Romania (32.9 %), The United Kingdom (30 %) and Denmark (25.8 %). Nuclear energy was used in 50 % of the EU-28 Member States. Lithuania has stopped producing nuclear energy in 2009. EU Member States with high nuclear energy production were France (80.9 %), Belgium (75.2 %), Slovakia (64.1 %), Sweden (49.4 %), Spain (42.6 %), Hungary (39.3 %) and Slovenia (38.5 %). Primary energy production from renewables in the EU-28 has increased by the 72.5 % during the 2004–2013 decade (Eurostat 2015).

The EU-28’s major primary energy producers from renewables were Germany (19 %), Spain (12 %), France (11 %), Italy (11 %) and Sweden (10 %). Renewable energy, promoted by the EU in particular for energy security and sustainability/decarbonisation reasons for almost two decades, constitutes the most indigenous form of energy, with imports (of biomass) constituting only 4 % of total renewable energy production. In 2012 the production of renewable electricity reached 799 TWh. Hydro power is the most important renewable electricity source and accounts for 46 % of renewable electricity generation in the EU, biomass 18 %, and wind and solar power 35 % (or 7 % of gross electricity production). As the share of wind and solar power grows, however, further modernisation of the grid and system operations will be necessary to ensure the electricity supply continues to be reliable. Nuclear powered electricity constitutes 13 % of the EU’s energy consumption, and 27 % of its electricity generation. 95 % of the fuel, uranium, is imported, from a variety of supplying countries (including Kazakhstan, Canada, Russia, Niger and Australia), for the EU’s 131 nuclear power plants (in 16 Member States, led by France, the UK, Sweden, Germany, Belgium and Spain). The Euratom Treaty set up a common supply system for nuclear materials, in particular nuclear fuel, established the Euratom Supply Agency to guarantee reliability of supplies and equal access of all EU users to sources of supply (In-depth study 2014).

**Fig. 1:**
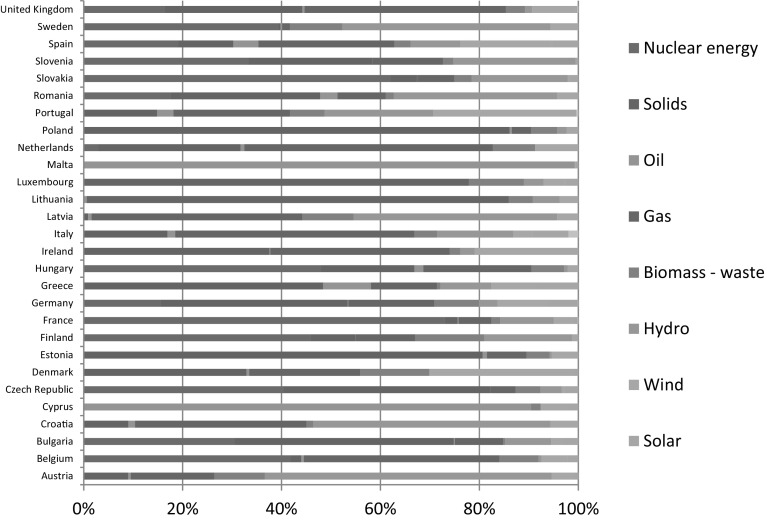
Energy-mix of EU Member States

The demand of the European countries for energy resources greatly exceeds their own resources therefore, there is a significant problem for the European economies in terms of dependence on the import of energy resources. The size of the import depends on the energy mix of the countries, as was presented in Fig. 1.

Import dependency shows the extent to which a country relies upon imports in order to meet its energy needs. It is calculated using the following formula (EEMS 2013):

1 $$ID = \frac{{_{{M_{j} \,-\, X_{j} }} }}{{GIC_{j} + Bunk_{j} }}$$

X is the export, M is the import, J is the energy product, GIC is the gross inland consumption, bunk is the consumption of international bunkers, unit = %.

Import dependence has been calculated for the following products: natural gas, crude oil, solid fuels (hard coal and derivatives, and lignite and derivatives) plus the total that is all of the above products together.

The EU countries are increasingly (but in a diverse manner, what has been provided later in this article) dependent on the import of hydrocarbons. The net energy import grows in the EU at a rate of several percent per year Table 2. According to the conservative estimates of the European Commission, until 2030 the increase of the import of energy resources in predicted, what means the increase of the energy dependence from the level of 54 to about 65 %, including the increase of the gas dependence to 84 % and on oil to approx. 93 %.

**Table 2:** Energy dependence of EU Member States in the years 2004–2013

	2004	2005	2006	2007	2008	2009	2010	2011	2012	2013
All products	50.2	52.2	53.6	52.9	54.7	53.7	52.8	54.0	53.3	53.2
Solid fuels	38.2	39.4	41.7	41.5	44.9	41.1	39.5	41.7	42.2	44.4
Total petroleum products	79.7	82.2	83.4	82.3	84.3	83.5	84.4	85.1	86.4	87.4
Natural gas	53.6	57.1	60.3	59.5	61.7	63.4	62.1	67.1	65.8	65.3

*Source * Eurostat, statistical books: Energy, transport and environment indicators (2015) edition

Among the member states there is a considerable variation in terms of energy dependence. The highest dependence in terms of oil, gas and coal in 2013 has been shown by Malta (104.1 %), Luxembourg (96.9 %), Cyprus (96.4), Ireland (89 %), Lithuania (78.3 %), Belgium (77.5 %) and Portugal (73.5 %). In contrast, most independent energy were: Estonia (1.9 %), Denmark (12.3 %), Romania (18.6 %), Poland (25.8 %), Netherlands (26 %), Czech Republic (27.9 %) and Sweden (31.6 %).

The highest level of energy dependence of the European Union countries occurs due to oil and in 2013 it amounted to 87.4 %. The following countries are totally dependent on the import of this raw material (100 %): Belgium, Bulgaria, Ireland, Cyprus, Latvia, Luxembourg, Malta, Finland and Sweden. The following countries import oil in over 90 %: the Czech Republic, Germany, Greece, Spain, France, Italy, Lithuania, Netherlands, Austria, Poland, Portugal, Slovenia. Denmark, as the only EU country in 2013 showed the negative rate of energy dependence on oil (−13.7 %), however, this level has definitely changed compared to 2004, when it amounted to (−115.9 %). Major changes also occurred in the UK, where in 2004 the UK also showed a negative rate of energy dependence on oil at the level of (−16.9 %) while already in 2013 it is the oil importer in 39.8 %. Member states also show the high dependence on the gas import, which level in 2013 was on average 65.3 %. 16 member states depends over 90 %, and only two countries were characterised by the negative level of energy dependence on gas, i.e., Denmark (−23.1 %) and Netherlands (−86.8 %). With regard to coal and oil, the level of dependence of the EU countries on the gas import increased to the greatest extent on the turn from 2004 to 2013 by 11.7 %. In the case of coal and its derivatives, in the years of 2004–2013 there was an increase of the dependence of the member countries in terms of this raw material by 6.2 % i.e. from the level of 38.2 % to the level of 44.4 %. The highest level of dependence on this raw material is shown by: the Netherlands (111.6 %), (Croatia 110.1 %), Luxembourg (100 %), Lithuania (99.7 %), Italy (96.2 %) and France (93.4 %), Portugal (95.4 %) Belgium (95.1 %). Only three EU countries are self-sufficient in terms of availability of this energy resources, i.e., the Czech Republic (−11.6 %), Estonia (−0.1 %) and Poland (−10.4 %) Table 3. Coal mining is a strategic sector of the Polish economy and plays a key role in ensuring the energy security of the country however, must be subject to a continuous process of restructuring in order to adapt to current market requirements (Burchart-Korol et al. 2014; Dubiński and Turek 2014; Krause et al. 2015; Bluszcz and Kijewska 2015; Kijewska 2016a, b; Blaschke and Gawlik 1999; Gawlik and Mokrzycki 2014; Brzychczy 2012; Kustra and Kubacki 2009; Kustra and Sierpińska 2008; Krzemień et al. 2013; Jonek-Kowalska 2015; Nawrocki and Jonek-Kowalska 2016; Strozik et al. 2016; Ranosz 2014).

**Table 3:** Energy dependence level of EU member states in 2013

	Solid fuels and derivatives	Total petroleum products	Natural gas
Austria	93.8	92.9	75.5
Belgium	95.1	102.0	100.5
Bulgaria	16.1	103.7	93.2
Croatia	110.1	77.1	31.8
Cyprus	100.0	101.0	0.0
Czech Republic	−11.6	96.3	100.2
Denmark	90.7	−13.7	−23.1
Estonia	−0.1	59.9	100.0
Finland	65.7	106.2	99.9
France	93.4	98.9	97.4
Germany	44.5	96.1	87.2
Greece	3.2	94.2	100.0
Hungary	29.5	83.9	72.1
Ireland	72.4	100.2	95.9
Italy	96.2	90.7	88.1
Latvia	88.8	100.4	115.6
Lithuania	99.7	93.2	100.0
Luxembourg	100.0	100.3	99.6
Malta	0.0	104.6	0.0
Netherlands	111.6	94.7	−86.8
Poland	−10.4	91.3	74.2
Portugal	95.4	97.2	101.5
Romania	18.9	47.0	11.9
Slovakia	80.6	88.5	95.6
Slovenia	19.4	95.8	99.6
Spain	70.3	97.4	98.6
Sweden	82.4	101.5	99.1
United Kingdom	82.0	39.8	50.1

*Source* Eurostat, statistical books: Energy, transport and environment indicators (2015) edition

The main direction of oil import to the EU countries in 2013 where Russia, which provided 33.5 % of the European Union demand. Norway covering 11.7 % of the demand and correspondingly Saudi Arabia (8.6 %) and Nigeria (8.1 %). These four countries in total covered as much as 61.9 % of oil used in the EU. The remaining countries from which oil was imported included Kazakhstan (5.8 %), Azerbaijan (4.8 %), Algeria (3.9 %), Iraq (3.6 %) and other countries.

The import of natural gas took place mainly from Russia (39 %), Norway (29.5 %), Algeria (12.8 %) and Qatar (6.7 %). These four countries covered as much as 88 % of the whole gas import in the EU. Other directions of gas import include: Nigeria with the share of (1.8 %), Libya (1.8 %), Trinidad and Tobago (0.8 %), Peru (0.5 %), Turkey (0.2 %) and other countries.

In relation to coal, the import takes place mainly from Russia (28.8 %), Colombia (22 %), the United States (21.8 %). These three countries together covered as much as 72.6 % of the demand. Other countries, from which the import is done, include Australia (7.3 %), South Africa (6.83 %), Indonesia 3 %, Canada 1.7 %, Ukraine 1.5 %, Norway 0.6 % and other countries (Eurostat 2015).

## Methodology and results

The issue of energy dependence of the European countries currently is an important research problem of economic, social, geo-political and economic sciences. The diversity of countries in terms of their energy potential is widely published in the literature.

However, there are no accurate comparative analyses, which were presented in the article. The energy needs of the countries can be met from different sources, hence also the problem of conducting the comparative analysis with traditional methods. The article used the multidimensional comparative analysis, the genesis of which comes from the use in taxonomic methods of the concept of the multidimensional object, which is a statistical unit, and in the article this will include the European Union member countries, which level of energy dependence has been described by the variables, such as the level of dependence on the oil, gas and coal as the main energy resources. The multidimensional comparative analysis, deals with methods and techniques of comparison and analysis of the multivariate objects. The multidimensional comparative analysis is the formally coherent set of statistical methods used for the purposeful selection of information on the elements of some community and detection of the regularities in the mutual relations of these elements. The multidimensional comparative analysis is an interdisciplinary method that uses achievements and methods used in other areas. The multidimensional comparative analysis is a method, where the analysis is carried out in stages and in different directions.

The first stage of the research involved the standardisation of the input data presented in Table 3 according to the formula (Morzy 2013)

2 $$z_{i} = \frac{{x_{i} - \bar{x}}}{{S_{x} }}$$

where:

$\bar{x}$ i *S*_*x*_ are respectively, the mean and standard deviation of the variable in the sample.

As a result of the standardisation, the arithmetic means of the variable takes the value of zero, and the standard deviation the value of one, what has been presented in Table 4 (Panek 2009).

**Table 4:** Data matrix after standardisation

	Solid fuels and derivatives	Total petroleum products	Natural gas
Austria	0.7663	0.2230	0.1004
Belgium	0.7977	0.5780	0.6159
Bulgaria	−1.1097	0.6443	0.4654
Croatia	1.1599	−0.3932	−0.8008
Cyprus	0.9160	0.5389	−1.4566
Czech Republic	−1.7785	0.3556	0.6097
Denmark	0.6915	−3.9348	−1.9329
Estonia	−1.5009	−1.0641	0.6056
Finland	0.0879	0.7418	0.6036
France	0.7567	0.4570	0.5520
Germany	−0.4240	0.3478	0.3417
Greece	−1.4212	0.2737	0.6056
Hungary	−0.7862	−0.1280	0.0303
Ireland	0.2496	0.5077	0.5211
Italy	0.8243	0.1372	0.3602
Latvia	0.6456	0.5155	0.9273
Lithuania	0.9088	0.2347	0.6056
Luxembourg	0.9160	0.5116	0.5974
Malta	−1.4985	0.6794	−1.4566
Netherlands	1.1961	0.2932	−3.2466
Poland	−1.7496	0.1606	0.0736
Portugal	0.8050	0.3907	0.6366
Romania	−1.0421	−1.5673	−1.2112
Slovakia	0.4476	0.0514	0.5149
Slovenia	−1.0300	0.3361	0.5974
Spain	0.1989	0.3985	0.5768
Sweden	0.4911	0.5585	0.5871
United Kingdom	0.4814	−1.8481	−0.4234
The arithmetic mean	0	0	0
The standard deviation	1	1	1

*Source* own elaboration

The second stage of the research included the selection of the agglomeration method. The agglomeration methods lead to the creation of the connection tree (i.e. dendrogram), which is the graphical illustration of the way and the hierarchy of connecting the objects due to the decreasing similarity between the objects included to the tree in the next stages and the objects previously included in the tree. The hierarchy of these connections allows you to determine the mutual position of the objects and groups of objects created on the next stages of the tree creation. The groups of similar objects create separate branches on this hierarchical tree. The tree hierarchy of the set of subjected O objects is defined as the ordered two <O;V>, where V is the matrix, which elements include the values of ultra-metrics between objects. The values of ultra-metrics indicate the distance, on which two objects meet on the connection tree. The starting point of the agglomeration methods is the assumption that each object constitutes a separate, one-element group (G_r′_ r = 1,2, …,z). Then, in the next steps, the groups of objects which are the most similar due to the values of the described variables are connected. The measure of this similarity includes the distances between the groups of objects. In the first step the distances between the one-element groups of objects G_1_, …, G_z_ are the elements of the input matrixes of the D distance. Objects are treated as the spatial objects. In the D matrix we are looking for the smallest distance between these groups of objects (Panek 2009):

3 $$d_{{rr^{\prime }}} = \mathop {\hbox{min} }\limits_{{ii^{\prime }}} \{ d_{{rr^{\prime }}} \}, \quad i = 1,2, \ldots ,n_{{r}} ;i^{\prime}=1,2,\ldots,n_{{r^{\prime}}};r,r^{\prime } = 1,2, \ldots ,z;r \ne r^{\prime }$$

where:

*d*_*rr*′_ is the distance of the r-th from the r′-th group.

**Fig. 2:**
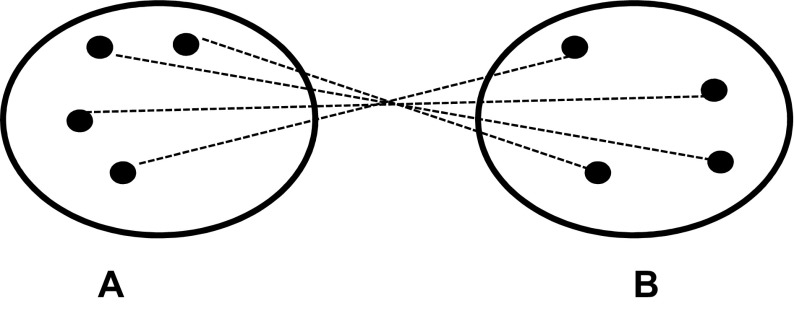
Distance between objects in two groups according to Ward method

The most similar objects are connected into one group, what causes the reduction of the initial group by one, starting the construction of the connection tree. Then the distances of the newly created group of objects are determined from all other groups of objects. These distances are placed into the D distance matrix into the spot of rows and columns corresponding to the objects (groups of objects) connected into one group. The procedure of connecting groups of objects is repeated until they create one group (that is one full connection tree has been created), i.e., n−1 times. After each stage of grouping the distance of the newly created group of objects is determined from the other groups of objects. These distances create a new matrix, which is current at the given stage of grouping, of distances with the increasingly smaller size (n−u) (n−u), where u—is the u stage of connection of the object groups. The general formula for determining the distances of the newly created group of objects G_r′′_, by connecting the groups of objects G_r_ and G_r′_, from other groups of objects G_r′′′_, while creating the connection tree has the following form (Panek 2009):

4 $$d_{r'''r''} = \alpha_{r} d_{r'''r} + \alpha_{r'} d_{r'''r'} + \beta d_{rr'} + \gamma \left| {d_{r'''r} - d_{r'''r'} } \right|$$

where:

$\alpha_{r} ,\alpha_{r'} ,\beta ,\gamma$-coefficients (parameters) of transformations different for various agglomeration methods. Specific agglomeration methods differ in methods of determining the distances between the objects. The most commonly used ones in practice include:—the method of the nearest neighbourhood (the method of the single binding), the method of the furthest neighbourhood (the method of full binding), the method of average connections) the inter-group means), the method of gravity, an which is one of the most popular ones and has been used in this article. The inter-group distances in the applied Ward method are presented in the Fig. 2.

In this method the distances between two groups of objects cannot be presented directly with the distances between the objects belonging to these groups. Two groups of objects while creating the tree of connections, at any stage, are connected into one group, so as to minimise the sum of squares of deviations of all objects from these two groups. This means that at any stage of connecting the groups of objects those groups are connected into one group, which as a result create the group of objects with the smallest diversification due to the variables describing them. The measure of this diversification is the error sum of squares criterion (ESS) formulated by Ward (1963) with the form of (Panek 2009):

$$ESS = \sum\limits_{i'' = 1}^{{n_{r''} }} {d_{{i''i''^{c} }}^{2} } \left( {O_{i''} \in G_{r''} ,\;O_{{i''^{c} }} = \overline{{O_{r''} }} \in G_{r''} } \right)\;\quad \quad \quad \quad \quad \quad (5)$$

where:

$d_{{i''i''^{c} }}$ is the distance of the i′′-th object belonging to the newly created r′′-th group from the centre of gravity of this group.

$$O_{{i''^{c} }} = \overline{{O_{r''} }} = \frac{1}{{n_{r''} }}\sum\limits_{i'' = 1}^{{n_{r''} }} {O_{i''} } \quad \quad \quad \quad \quad \quad \quad \quad \quad \quad \quad (6)$$

In the method of Ward the parameters of transformations in 4 formula have the values:

$$\begin{aligned} \alpha_{r} = \frac{{n_{r} + n_{r'''} }}{{n_{r} + n_{r'} + n_{r'''} }} \hfill \\ \alpha_{r'} = \frac{{n_{r'} + n_{r'''} }}{{n_{r} + n_{r'} + n_{r'''} }} \hfill \\ \beta = \frac{{ - n_{r'''} }}{{n_{r} + n_{r'} + n_{r'''} }} \hfill \\ \gamma = 0 \hfill \\ \end{aligned}$$

The third stage was the selection of the distance measure between the objects. The research has used the square of the Euclidean distance as the measure of distance between the objects according to the formula (Giudici 2003):

7 $$D_{XY} = \sum\limits_{k = 1}^{m} {\left( {x_{ik} - x_{jk} } \right)}^{2}$$

where:

$X = \left( {x_{i1} , \, x_{i2} , \, \ldots , \, x_{im} } \right)$, $Y = \left( {x_{j1} , \, x_{j2} , \, \ldots , \, x_{jm} } \right)$ are two *m*-dimensional objects.

**Fig. 3:**
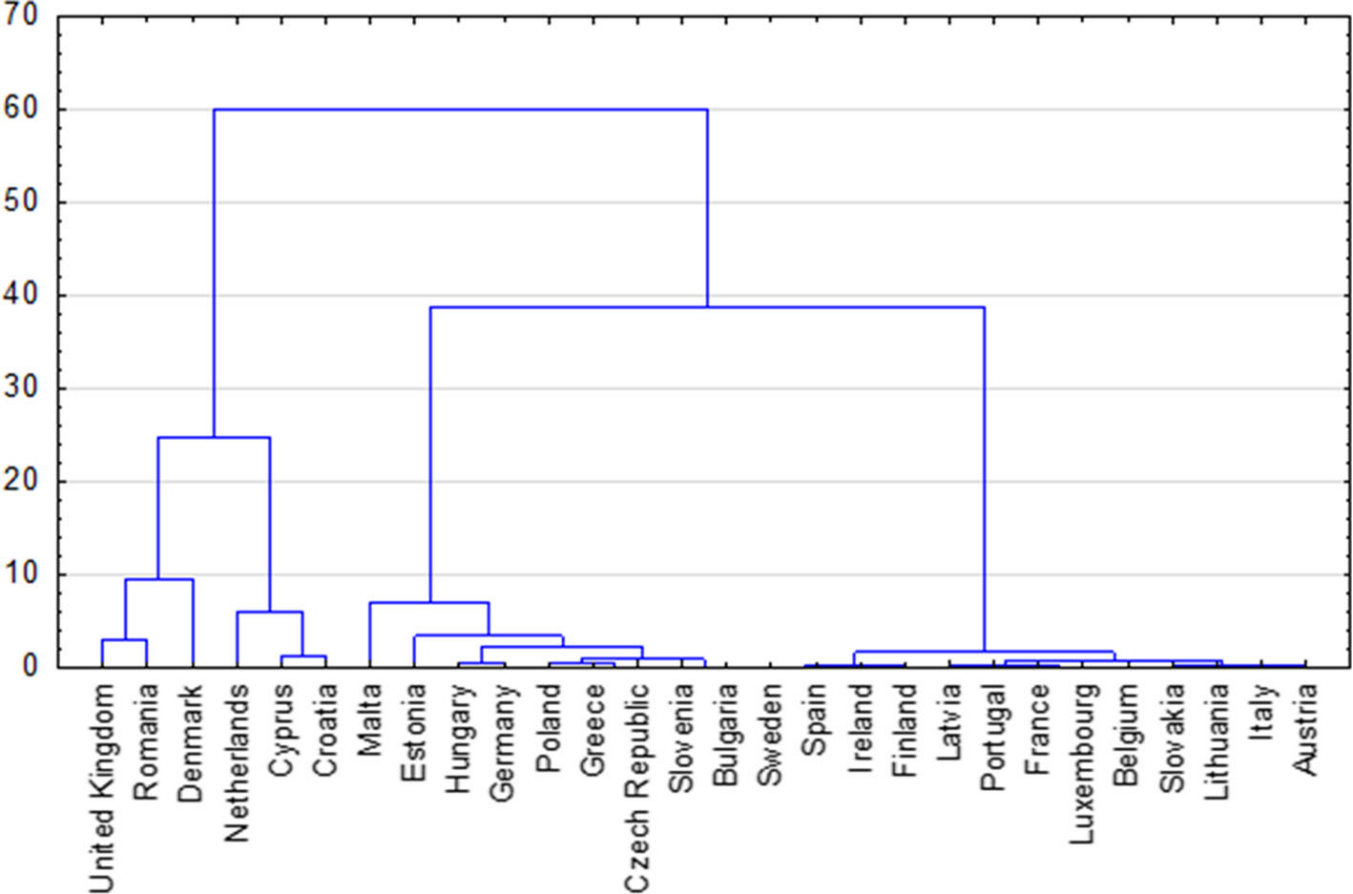
Dendrogram of grouping EU countries according to energy dependence level

The results of the agglomeration according to the Ward method using the square of the Euclidean distance as the measure of distance between the European Union member states led to the graph of the connection tree presented in Fig. 3.

**Fig. 4:**
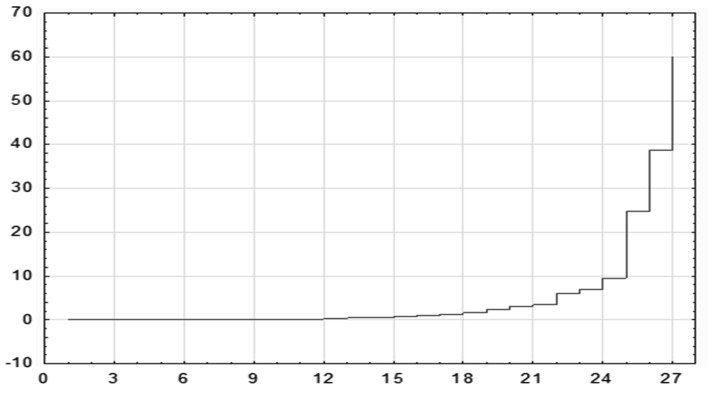
Graphs of the course of agglomeration for energy dependence by Ward’s method

Based on the dendrogram graph and based on the binding distance graph (presented in the Fig. 4) in relation to the stages of binding it is possible to indicate seven groups of homogenous countries in terms of the level and quality of the energy dependence.

The fourth stage of the research included the optimisation of the initial grouping.

In order to optimise the initial grouping of objects into seven homogenous groups, the method improving the well-being of the initial grouping has been used by moving—transferring the objects, that is countries between the preliminary groups from the point of view of the defined criterion of the well-being of grouping. The k-means method has been selected as the method of optimisation.

In the k-means method the aim is to find the division of a set of objects *D* between the *k* clusters $C_{1} , \, C_{2} , \, \ldots ,C_{k}$, of means that minimizes the criterion function *e(k)*. In the basic version of the algorithm the criterion function that is minimized, is the sum of the squared error (Larose 2005):

$$e(k) = \sum\limits_{i = 1}^{k} {\sum\limits_{{p_{j} \in C_{i} }} {dist\left( {p_{j} ,m_{i} } \right)^{2} \quad \quad \quad \quad \quad \quad \quad (8)} }$$

where *p*_*j*_ is the point in *R* ^*m*^ space that represent object *p*_*j*_, *m*_*i*_ the mean of cluster *C*_*i*_, *dist(p*_*j*_, *m*_*i*_ *)* the Euclidean distance (norm *L*_*2*_) between object (point) *p*_*j*_ and mean (centre) *m*_*i*_ of the nearest cluster *C*_*i*_.

Algorithm can be described as follows (Han and Kamber 2006; Hastie et al. 2009; Mirkin 2011):

A data set containing *n* objects is given and the number of clusters *k* is assumed (Fig. 4).

1. Arbitrarily choose *k* objects from *D* as the initial cluster centres;
2. Repeat steps (a) and (b) until there are changes in the allocation of objects to clusters:
  - (re)assign each object *p*_*i*_ *∈* *D* to the cluster *C*_*i*_, to which the object is most similar, based on the mean value of the objects *p*_*i*_ in the cluster *C*_*i*_;
  - update the cluster means, i.e., calculate the mean value of the objects for each cluster.

The results of the grouping optimisation are presented in Table 5.

**Table 5:** Optimisation of grouping with the k-means method

Country	Distances from centre of cluster
Cluster I	
Denmark	0
Cluster II	
Romania	0
Cluster III	
Croatia	0.476035
United Kingdom	0.476035
Cluster IV	
Cyprus	0.527802
Netherlands	0.527802
Cluster V	
Malta	0
Cluster VI	
Estonia	0.708067
Germany	0.483398
Czech Republic	0.365788
Hungary	0.365564
Poland	0.362641
Bulgaria	0.313597
Slovenia	0.199513
Greece	0.181967
Cluster VII	
Finland	0.357612
Austria	0.297258
Spain	0.236240
Italy	0.229430
Slovakia	0.226777
Latvia	0.225507
Ireland	0.215214
Lithuania	0.203003
Luxembourg	0.189651
Belgium	0.151599
Portugal	0.124113
Sweden	0.111389
France	0.090725

*Source* own elaboration

**Fig. 5:**
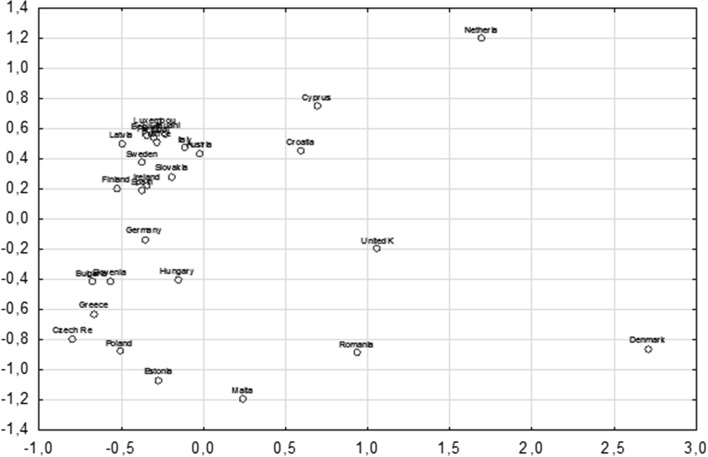
EU member states in the two-dimensional space

As a result of the analysis seven clusters were formed containing the uniform elements due to the energy dependence of the studied raw materials, i.e., oil, gas and coal. The system of objects in the two-dimensional space is presented in the scattering graph in the two-dimensional space in Fig. 5.

The configuration of the objects indicates that Denmark is the object that is the most projecting one in the two-dimensional space and creates the one-element first cluster. The second cluster is created by Romania. The third cluster is created by Croatia and the United Kingdom, their distance from the centre equals 0.476035. The fourth cluster is created by Cyprus and the Netherland, their distance from the centre equals 0.527802. The 5th cluster is created by Malta. The sixth cluster is created by eight countries. The most typical for this cluster is Greece—the smallest distance from the centre of cluster (0.181967), then Slovenia with the distance of (0.199513), Bulgaria (0.313597), Poland (0.365564), Hungary (0.365564) and Czech Republic (0.365788). Objects that significantly atypical include Germany with the distance from the centre of cluster (0.483398) and the furthest object, which is Estonia (0.708067). The seventh cluster consists of countries, such as: Finland, Austria, Spain, Italy, Slovakia, Latvia, Ireland, Lithuania, Luxembourg, Belgium, Portugal, Sweden and France. The most typical country in this focus includes France with the distance from the centre of cluster (0.090725), while the most atypical element in the cluster involves Finland with the distance (0.357612) what has been presented in Table 5 and Fig. 5.

## Conclusions

Currently the issue of the sustainable development is one of the major problem of the European Union economy (Hąbek 2012, 2014; Hąbek and Wolniak 2016). The access to energy is one of strategic importance for the development and existence of the societies, hence the issue to ensure its uninterrupted deliveries is of priority importance today for the economies of the EU member states. The strategic position of energy means writing the issue of energy security in the discourse on the national interest. The issue of the import dependence of the member states bears the need to prepare and design the strategic solutions in the energy policy in the Union. Outlines in the article is the issue of using the taxonomic methods for the construction of models and tools enabling the selection of countries similar due to the energy conditions, which brings potential possibilities in the sphere of building the models for grouping elements into uniform sets with the possibility of their use for creating long-term strategies taking into account similar structures of countries grouped in the exemplary clusters. At the same time it should be stressed that there is a wide range of applicable mathematical models, which can be used in this type of issues. The task of the author was to present the selected model in order to indicate one of many possible solutions with the provision that many aspects of the subject matter has not been fully exhausted.
